# Supplementary material for: Are carbon emissions trading and green financial instruments synergistic? -Comprehensive quantitative research based on content analysis
Source: PLoS One. 2024 Mar 7;19(3):e0298601. doi: 10.1371/journal.pone.0298601 (PMC10919601; doi:10.1371/journal.pone.0298601)
Supplement: S1 Data — (ZIP) [file pone.0298601.s001.zip › Data/地级市Z_F工作报告.zip!/╦─┤¿/├╝╔╜╩╨/└┤╫╘╣1⁄2╓┌║┼í╛┬φ┐╦╩2╛▌═°í┐_2015.docx]

各位代表：

我代表市人民政府，向大会报告工作，请予审查，并请政协委员提出意见。

2014年工作回顾

2014年，是眉山经济社会发展极其艰难和极具挑战的一年。我们在省委、省政府和市委的坚强领导下，在市人大、市政协的监督支持下，始终保持发展定力，全力以赴稳增长；始终坚持改革创新，贴身紧逼调结构；始终坚守为民底线，千方百计惠民生，较好地完成了市三届人大五次会议确定的目标任务。

一年来，全市经济社会发展呈现三大特点：

——在综合施策中稳增长，实现了换挡不失速的稳健发展。全市地区生产总值（GDP）944.89亿元，增长10.1%，居全省第4位；全社会固定资产投资922.98亿元，增长22.1%，居全省第5位；规模以上工业增加值增长10.2%，居全省第8位；社会消费品零售总额319.71亿元，增长12.9%，居全省第12位；民营经济增加值增长11.3%，居全省第3位。

——在扩大增量中调结构，实现了转型不失衡的提质发展。三次产业结构优化为16.1:57.1:26.8；战略性新兴产业占工业经济比重达19%，提高了2.2个百分点；第三产业增加值增长11.2%，居全省第3位；地方一般公共预算收入75.2亿元，同口径增长15.6%，占GDP比重为8%，居全省第6位；民间投资占全社会投资比重达74.4%；招商引资到位资金连续保持全省第2位；超额完成省下达的节能减排任务。

——在利民富民中办实事，实现了保障不失位的惠民发展。十大民生工程155个项目全面完成，全市全部财政支出中81%用于民生；城镇居民人均可支配收入24135元增长10.2%，增速居全省第5位；农民人均纯收入10433元增长11.8%，总量保持全省第4位；城镇新增就业3.1万人；减少贫困人口4.94万人。

一年来，我们围绕“五个主题年”，主要做了以下工作：

大力推进产业发展，转型升级步伐加快。产业结构不断优化。高端成长型产业和新兴先导型服务业加快发展，新增规模以上工业企业54户，万元工业增加值能耗预计下降10%。现代服务业提速发展，消费市场持续繁荣，新增限额以上商贸企业236家，实现旅游收入180.65亿元。金融机构存款余额1253.85亿元，比年初增长12.45%，贷款余额625.17亿元，比年初增长17.52%。粮食总产量稳定在160万吨以上，粮食购销全省第一。质量强市深入推进，新增中国驰名商标、中国地理标志商标各1件，新增四川名牌16个。产业能级不断提升。产业集聚效应明显增强，彭山经开区、甘眉园区、金象园区等13个工业园区实现产值850.3亿元，占工业经济比重的67.1%。深入实施创新驱动战略，新增国家高新技术企业16家，实施重大科技成果转化30项，完成技改投资255.61亿元，淘汰落后产能企业37户。高端产业和产业高端成为发展的主方向，机械装备、化工等6大优势产业不断壮大，实现主营业务收入1047.4亿元，增长19.6%。产业模式不断创新。传统产业与现代产业相互渗透，实现电子商务网络零售交易24亿元。工业化与信息化深度融合，“智慧园区”示范建设深入实施。都市近郊型现代农业加快发展，现代畜牧业深化试点圆满完成，三大现代农业园区完成投入13.5亿元，现代农业绩效考核全省第一。成功举办第六届中国泡菜展销会并上升为国家展会。成功荣获2016年第九届中国竹文化节主办权。

大力推进全域统筹，城乡面貌显著提升。城乡建设快速推进。岷东新区加快建设，东坡岛开发、一环路改造推进有力，东坡城市湿地公园、“两宋荣光”眉州大道、东坡宋城I期、苏洵公园、苏辙公园等相继建成。完成城镇建设投入184.1亿元，新增城镇面积16.46平方公里。强力推进县城建设，大力实施“百镇建设行动”，房地产业健康有序发展，城镇综合承载能力明显增强。交通建设推进有力，成德绵眉乐高铁、遂资眉高速、眉山交通中心、岷江汉阳航电工程建成投运，成都经济区环线眉山段开工建设，仁沐新高速仁寿段加快建设，“四箭齐发”、大峨眉国际旅游西环线加快推进，完成综合交通投入121.8亿元。城乡体系日臻完善。中心城区“166”控制区总体规划编制完成，仁寿、洪雅、丹棱、青神县城市总体规划修编顺利推进，128个乡镇规划全面完成。坚持“四种模式”“四个转变”，全年农民新转变为城镇居民15.83万人、产业工人8.98万人、三产经营（从业）者3.8万人、现代农业业主1616户。城乡公共服务设施不断完善，完成文化类设施投入4819万元。城乡环境明显改善。深入推进城乡环境治理，“五乱”整治深入开展，“大城管”工作格局全面构建。东坡湖综合整治全面完成，“两岸交相辉映、湖水碧波荡漾”的目标基本实现。农村生活垃圾处理眉山模式全国推广，丹棱“环境优美示范县城”创建工作全省第一。“绿海明珠”“千湖之城”“百园之市”加快推进，新增水域面积32.5平方公里，森林覆盖率45.97%。国家级园林城市、省级文明城市成功创建，省级双拥模范城创建通过验收，洪雅通过国家级生态县技术评估，黑龙滩成功创建国家4A级景区。

大力推进改革开放，发展动力日益强劲。综合改革推进有力。经济体制改革顺利推进，社会活力全面激发，新增各类市场主体17107个，民间投资增长17.9%，民营经济增加值实现546.16亿元。行政体制改革破冰前行，新一轮政府机构改革顺利实施，事业单位分类改革加快推进，彭山撤县设区工作全面完成，3个乡实现撤乡设镇。投融资体制改革成效明显，成功发行城投债券14亿元，推进10家企业在上海、成都股权交易中心挂牌。统筹城乡改革成效明显，农村土地确权颁证深入推进，土地流转率达到30%，新增各类农业经营主体2279个。对外开放占据高地。投资促进工作再上台阶，成功引进法国达能、中建钢构、中联重科等世界500强、中国500强及投资10亿元以上项目25个，招商引资到位资金650亿元，西博会签约金额全省第二。对外贸易平稳发展，新增外贸企业53家，进出口总额3.3亿美元，增长15.33%。成功承办中韩自贸区第11轮谈判。区域合作纵深拓展。成眉合作成效明显，天府新区建设强力推进，完成投资103亿元，新落户项目23个。雅眉合作全面开启，成功签署框架协议和专项协议。甘眉合作深度发展，甘眉园区新增规模以上企业7户，争取留州电量24亿千瓦时。

大力推进社会建设，民生福祉持续改善。民生实事成效明显。棚户区改造首批争取国家开行贷款24.7亿元，占全省总额的1/13，保障性住房开工率全省第二。社会保障全面加强，城乡低保标准进一步提高，社会养老服务体系加快建设，城镇居民“零就业”家庭保持动态为零，农村五保集中供养率全省第一。残疾人创业就业基地建设全省第一，新农合参合率99.94%全省第二。“4·20”芦山灾后恢复重建成效显著。社会事业全面进步。教育事业加快发展，学前教育毛入园率89%，义务教育招生改革经验全省推广，成功组建眉山市职业教育集团，四川师范大学成都学院正式招生，四川城市职业学院、成都信息工程学院天府校区、四川科技职业学院、成都艺术职业学院、成都七中实验东坡学校等开工建设。体育事业蓬勃发展，我市参加青奥会、省运会分别获2枚和35枚金牌。现代公共文化服务体系加快建设，文化立市“123”工程深入推进，“五馆一中心”完成深度规划，第四届中国西部书法篆刻作品展成功举办。卫生计生事业全面加强，实现每个县有1所二甲以上综合医院，公立医院药品加成全面取消，省内异地就医实现即时结算，乡镇卫生院标准化建设达标率全省第一，单独两孩政策全面实施。公积金管理、市档案馆馆藏档案数字化率全省第一。第三次全国经济普查工作全面完成。国防建设、民族宗教、老龄事业、妇女儿童、史志研究等工作取得新成绩。社会大局和谐稳定。深化“平安眉山”建设，依法治市“八大体系”深入推进，现代社会治理体系初步构建。切实加大对违法犯罪活动的打击力度，群众安全感、满意度稳步提升，网格化服务管理工作经验在全省推广，“大调解”工作全省领先，“诉非衔接”改革试点终期评估全国第一。完善社会稳定风险评估制度，深化维稳跟着项目走，群众来访“四个当天”办理制度成效明显。食品药品监管全面加强。安全生产形势持续好转，主要考核指标连续4年下降，无重大及以上安全事故发生。

大力推进自身建设，法治政府全面加强。依法行政深入推进。法制宣传亮点纷呈，“六五”普法中期考核全国先进，“法律七进”工作经验全省交流。行政决策科学民主，坚持“三重一大”事项集体决策。行政执法公正透明，全面规范行政执法裁量权。行政监督保障有力，不断完善政府内部层级监督，强化部门专项监督，审计工作连续3年被评为全省先进。认真办理人大代表建议、政协提案，满意率和基本满意率达100%。政府职能切实转变。大力实施简政放权，推动放管结合，全年取消和下放行政审批36项，清理非行政审批69项。扎牢扎密制度笼子，着力构建长效机制，制定出台规范性文件27个。充分激发市场活力，清单管理制度初步建立，大众创业、万众创新的局面逐步形成。创新政务服务星级管理，率先在全省建成“一目录、两平台、一大厅”，政府采购推行网上竞价，公共资源交易规范高效。作风建设全面加强。深入开展党的群众路线教育实践活动，“四风”问题得到有效整治，市本级“三公”经费下降10.4%，政府公文减少12.78%，清退整改超标办公用房11000平方米。扎实开展正风肃纪专项行动，大力整治“庸懒散浮拖”，查处涉及“四风”问题案件160件170人，通报曝光典型案件59起。

各位代表！

发展凝聚心血，奋斗伴随艰辛。过去一年，在多重困难集中显现、多重风险相互交织的情势下，我们用坚韧和顽强顶住了经济下行的巨大压力，用勤劳和智慧破解了前进道路上的瓶颈障碍，用拼搏和汗水赢得了加快发展的丰硕成果，“稳”的基础更加牢固，“进”的态势积极向好。成绩来之不易，这是省委、省政府和市委坚强领导的结果，是市人大和市政协监督支持的结果，是全市人民团结奋斗的结果。在此，我谨代表市人民政府，向历任老领导、老同志，向全市人民、解放军驻眉部队和武警、消防官兵、政法干警，向关心支持和参与眉山建设发展的社会各界人士，表示衷心的感谢并致以崇高的敬意！

在肯定成绩的同时，我们也要清醒地看到发展中面临的困难和问题。经济总量不大、产业支撑不强、发展滞后不足，仍是眉山发展的最大问题；产业结构不优、主导产业不突出、产业粗放低效，仍是眉山发展的最大差距；重大影响项目缺乏、资源环境约束加大、行政效能不高，仍是眉山发展的最大瓶颈；社会矛盾增多、系统风险集聚、安全形势严峻，仍是眉山发展的最大矛盾。对上述问题，我们将高度重视，认真加以解决。

2015年重点工作

2015年是全面完成“十二五”规划的收官之年，也是本届政府任期攻坚之年。做好今年政府工作，意义重大，任务艰巨。我们要认真贯彻落实党中央国务院、省委省政府和市委决策部署，主动适应新常态，紧紧围绕“八大攻坚任务”，坚持内需拉动、产业带动、创新驱动，从需求方面施策，从供给方面发力，均衡拉动经济增长，努力实现经济社会健康持续发展，奋力走在全省加快发展前列。

全市经济社会发展的主要预期目标为：地区生产总值增长8%；规模以上工业增加值增长8.5%；全社会固定资产投资完成800亿元；地方一般公共预算收入同口径增长8%；城镇居民人均可支配收入增长9%；农民人均纯收入增长10%；社会消费品零售总额增长11.5%；城镇化率提高1.7个百分点；居民消费价格涨幅控制在2%以内；城镇登记失业率控制在4.4%以内；人口自然增长率控制在5‰以内；全面完成省下达安全生产和节能减排任务。

需要特别报告和说明的是，这些目标是全市经济发展的平均值，不是最低值，是预期目标，不是约束指标。考虑我市经济体量即将突破千亿，实现这样的中高速增长仍然催人奋进、非常不易。在新的一年里，我们务必坚持既抓紧转又加快赶、既讲平衡又求突破、既为当前又利长远，能快就不慢，能跑就不走，能跳就不站，为全面实现各项目标而加倍努力。

实现上述目标，要突出抓好以下30项重点工作。

一、突出深化改革，激发内在活力

改革是发展的制胜法宝。我们将以深化行政审批制度改革为突破口，协调推进各领域改革，最大限度激发市场和社会活力，为全市经济社会发展注入新动能。

1.深化行政体制改革。以“减、放”为原则，以“不减、不放”为特例，纵深推进简政放权、放管结合，全面取消非行政许可审批事项，实行省市县联动审批。深入推进商事制度改革，营造宽松平等的市场准入环境。积极推进政府购买公共服务，有序推进事业单位分类改革、公务用车制度改革。

2.深化经济体制改革。深化金融体制改革，大力支持实体经济发展。着力培育金融市场主体，实现眉山农商行和仁寿、青神、丹棱农商行挂牌营业。深化财税体制改革，推动“营改增”扩面，全面推行预算改革，规范政府债务管理。启动不动产登记，进一步理顺和完善国有资产监督管理体制。创新投融资体制，加快政府投融资平台转型发展，推广使用PPP模式，积极发展混合所有制经济。支持民营经济加快发展，民营经济增加值增长8.5%，继续保持全省领先。

3.深化农村综合改革。以放活土地经营权为突破口，深化农村土地制度改革，加快土地确权颁证。积极发展适度规模经营，建立农村产权流转交易综合服务信息平台。创新农业经营体系，新增现代农业业主1500户、家庭农场100个、农民合作社50个、产业化龙头企业5户。加快彭山全省农村综合改革试点工作，力争在放活土地承包经营权、健全社会服务体系、农村金融制度等重点领域、关键环节取得突破。

4.深化社会领域改革。深化教育领域综合改革，引导民办教育机构规范发展。深化科技体制改革，加大科技金融创新力度。深化医疗卫生体制改革，鼓励社会资本进入医疗卫生领域，推进县级公立医院综合改革试点，实施分级诊疗制度。深化文化体制改革，推进文化大发展大繁荣。深化户籍制度改革，有序推进农业转移人口市民化。

二、突出项目投资，增强经济动力

抓发展重点在抓项目，稳增长关键在稳投资。我们将坚持项目总抓手、投资主驱动，引进实施一批具有基础性、先导性、支撑性的重大项目，不断增强经济发展动力。

5.强力推进招强引优。围绕世界500强、中国500强、行业领军企业，引进一批引领型、补链型、核心型高端产业项目；围绕战略性新兴产业，引进一批创新能力强、税收贡献高、资源占有少的优质企业；围绕优势产业，在上下游、左右链、曲线两端补足配套项目，形成中小企业铺天盖地、大型企业顶天立地的格局。精心组织“三走一请”“东坡味道”全国行等重大招商活动，招商引资到位资金566亿元以上，引进世界500强、中国500强或投资10亿元以上项目20个以上。

6.强力推进产业项目。大力推进汇源果汁生产基地、西南IT产业园及电子商务基地、富生电器等53个重点工业项目建设，完成投资88亿元。加快建设七里坪、黑龙滩长岛、老峨山、彭祖山等90个重点三产项目，完成投资180亿元。全面推进18个省级重点项目和100个市级重点项目，加快实施410个投资千万元以上“四个一批”项目。

7.强力推进基础建设。加快推进“四箭齐发”和成都经济区环线眉山段全线全段全面开工建设，分别完成投资21.6亿元和24亿元。全面推进成昆铁路扩能改造眉山段、仁沐新高速仁寿段、大峨眉国际旅游西环线、眉彭大道等项目，完成综合交通投资90亿元。加快推进植物园、生活垃圾焚烧发电等重大城市基础设施项目建设。抓好东坡宋城、苏母公园和蟆颐观等开发建设与改造，充实文化元素，打造城市文化地标。

三、突出转型升级，提升经济实力

转型才能更好发展，后发更要高点起步。我们将坚持产业兴市不动摇，坚持高端产业和产业高端融合互动，坚持传统优势产业和战略性新兴产业并重发展，努力在转方式中优存量，在调结构中扩增量。

8.加快工业提质增效。坚持聚焦工业、真抓工业、大干工业，深入推进百亿产业、百亿园区、百亿企业“三百工程”。加快推进新一代电子信息技术、新能源、高端装备制造和生物医药四大新兴产业，战略性新兴产业占比达到21%。加大传统产业技术改造和兼并重组力度，全年完成技改投入256亿元。坚持“一园一主业，园区有特色”，推动园区产业整合、上档升级。加快培育大企业大集团，力争金象赛瑞、千禾味业上市，新增规上企业30户。

9.培育壮大第三产业。突出五大新兴先导型服务业发展，加快发展第三方物流、现代保险、融资租赁、信息技术、服务外包等生产性服务业，大力发展养老、健康、文化、体育等生活性服务业。促进服务业与工农业协调发展、跨界发展、创新发展，推动电子商务、互联网金融、快递、文化创意等产业融合发展。第三产业增加值增长9%。

10.转变农业发展方式。依托“两城三园七片”，大力发展都市近郊型现代农业。继续做大做强泡菜、奶业、竹编等特色优势产业，力争泡菜销售收入达130亿元，鲜奶产量达15万吨，新建改造竹海基地2万亩。加快建设现代畜牧经济强市，主要畜禽规模化养殖比重达72%以上。积极鼓励种植业园区化、养殖业小区化，三大现代农业园区完成投资10亿元。加强以山水田林路为重点的农业基础设施建设，建设高标准农田示范区10万亩。严守耕地红线，粮食产量保持在160万吨以上，深入推进“粮安工程”，打造全省粮食流通产业强市。

11.加快发展旅游经济。加快推进江湾神木园、大雅堂、柳江古镇、中国泡菜城创建国家4A级景区，七里坪创建国际抗衰老健康产业试验区和国家级旅游休闲度假区，主动对接和融入全省旅游精品线路，完成旅游项目投入60亿元，实现旅游总收入194亿元。推进全市13个旅游集镇和14个乡村旅游片区建设，实现乡村旅游收入30亿元。

12.强化创新支撑引领。更加注重科技进步和自主创新，加快构建以企业为主体、政产学研用协同创新机制。推进科技与金融融合，加强科技企业孵化器、工程技术中心、产业联盟等创新载体建设，完善科技重大专项实施机制。加大高端人才引进，激励科技人才创新创业。全年技术创新投资增长15%，实施高新技术及战略新兴产品项目60项，新增年销售收入亿元以上高新技术企业或企业集团15家，新增国家高新技术企业5家、省级工程技术中心1个。

四、突出重点区域，发挥辐射引力

重点区域是推动发展的龙头，是追赶跨越的引擎。我们将创新机制、汇聚资源、集中力量，加快打造新的经济增长极，努力形成区域带动、点极共兴、竞相发展的良好局面。

13.天府新区“一马当先”。抢抓天府新区上升为国家级新区机遇，重构产业发展、重构体制机制、重构规划对标，打造眉山经济发展的重要引擎。大力培育高端装备制造、现代物流、精细化工、新一代电子信息产业集群，确保全年新引进5亿元以上项目8个，世界500强或国内行业100强企业2户，招商引资到位资金80亿元，实现产值380亿元以上。规划建设环天府新区，推动全域天府新区建设。

14.工业新城“七驾齐驱”。优化七大园区的主导产业，机械装备、日用化工、新型建材等传统优势产业实现增加值增长9%，新一代电子信息技术等战略性新兴产业实现增加值增长9.5%，完成投资150亿元，销售收入突破500亿元。加快上档升级，推动眉山经开区创建国家级经开区。科学合理确定总部经济区建设项目，全面启动总部经济区项目策划、控制性详规编制。

15.县域经济“六极共振”。深化“两扩两强”改革试点，激发六区县经济发展活力。坚持因地制宜、分类指导，突出地域特色和资源优势，做实做强县域产业，确保在全省县级经济综合评价中晋位升级。深入实施多点多极支撑发展战略，支持县域开发区和工业集中区加快发展，完成投资220亿元，实现工业增加值增长9%，建设一批宜居宜业的新型小城镇和富有活力的经济强镇。

16.区域合作“四城联动”。深入推进成眉、甘眉、雅眉合作，加快推进成眉工业集中区、成眉石化园区、甘眉园区建设，大力推进雅眉交通设施、旅游资源、要素市场协调发展。积极融入国家和省对外开放战略布局，抢抓国家建设“一带一路”、长江经济带的历史机遇，深化全方位开放合作，争取纳入国家战略规划的重要节点城市，密切与周边地区经济交流，大力发展开放型经济。

五、突出城乡统筹，释放发展潜力

城镇化是经济增长的重要支撑。我们将深入实施“两化互动，城乡统筹”发展战略，促进公共资源均衡配置，加快城乡协调发展、协同发展、共同发展。

17.始终坚持布局优化。继续优化经济空间布局，做大做强“一体两翼三带”，努力构建眉山经济发展新地图。抢抓彭山撤县设区的历史机遇，加快建设东坡、彭山城市版块经济，推进彭山区与东坡区同城发展，努力打造“两区双轮”的现代都市经济带。抢抓成德绵眉乐高铁、遂资眉高速、岷江汉阳航电建成投运和成都二机场规划建设的历史机遇，加快谋划引进一批新兴产业和重大项目，努力打造“两高双港”的现代枢纽经济带。加快推进天府新区青龙、视高两大区域建设，努力打造“两极双核”的现代产业经济带。

18.始终坚持聚焦城镇。以新型城镇化建设为总揽，扎实推进以人为核心的城镇化。进一步完善城镇规划体系，启动新一轮眉山城市总体规划修编。大力实施“十大工程建设计划”，不断完善城镇基础设施建设，完成投资28亿元。促进房地产业和建筑业健康有序发展，完成房地产开发投资150亿元。强力推进岷东新区建设，完成投资13亿元。加快推进东坡岛和北部郊区建设。高水平推进县城建设，提升城镇发展水平。继续抓好“百镇建设行动”，坚定不移做优小城镇，大力推进10个国家级重点镇、13个省级试点镇和22个市级特色镇建设。

19.始终坚持城乡一体。加快推进“四种模式”“四个转变”，推进城市基础设施向农村延伸，城市公共服务向农村辐射，城市现代文明向农村传播，力争全年农民转变为城镇居民14.2万人、产业工人8.8万人、三产经营（从业）者3.1万人、现代农业业主1500户。统筹“物的新农村”和“人的新农村”，建成幸福美丽新村200个以上。成片推进新农村建设，确保3个省级新农村建设成片推进示范县和7个市级新农村示范片全面完成年度目标任务。加快推进“1+6”村级公共服务体系建设，创建一批“美丽乡村”示范村庄、国家级和省级生态文明乡村。

20.始终坚持建管并重。加强城市管理综合执法机构建设，提高执法和服务水平。健全数字城管工作机制，提升城市管理网格化、信息化和标准化水平。建立健全城镇基础设施管理体制机制，抓紧建立城镇基础设施电子档案，着重加强城镇地下管网综合管理。积极创建第四届四川省文明城市。

六、突出生态文明，彰显环境魅力

向污染宣战，既是攻坚战，更是持久战。我们将坚持不懈地保护生态环境，源头严防、过程严管、后果严惩，治标治本多管齐下，朝着天蓝地绿水净的目标不断前进。

21.严守生态保护红线。严格环境准入，优化项目环评审批。加强环境监测预警和监管执法，深入实施环保专项行动，PM10浓度下降2%。狠抓重点区域、重点流域污染整治，严厉打击环境违法犯罪行为。开展企业环境信用评价，建立环保“黑名单”制度。深入推进城乡环境综合治理，加强农村环境保护。

22.坚持不懈节能减排。落实节能减排目标责任制，加快淘汰落后产能，化解过剩产能，力争单位工业增加值综合能耗下降3%以上、工业固废综合利用率提高到85%，工业废气排放量控制在指标以内。

23.努力打造生态品牌。强力推进“绿海明珠”建设，森林覆盖率保持46%以上，新增城市绿地1万亩。强力推进“千湖之城”建设，恢复和新增水域面积25平方公里，争创全国水生态文明试点市。强力推进“百园之市”建设，新建和改造各类公园10个，“四大文化主题公园”全面建成。

七、突出民生改善，优化保障能力

民生问题是政治问题，是社会问题，也是发展问题。我们将把民生改善作为一切工作的出发点和落脚点，搞好“基本民生”、保障“底线民生”、关注“热点民生”，推动人民生活水平不断提高。

24.扎实办好民生实事。继续实施十大民生工程，办好20件民生大事，加大民生投入，确保全市全部财政支出中用于民生的比重稳定在80%以上。实施精准扶贫，减少农村贫困人口4万人。大力抓好“百万安居工程”，强力推进危旧房棚户区改造，提高货币化安置比例，改造棚户区12738户，建设保障性住房2906套，农村危房改造2000户，确保公租房房源的30%以上定向分配给农民工。

25.提高社会保障水平。稳步提高城乡低保及优抚对象抚恤补助标准，完善城乡居民大病保险制度，提升城乡居民保障水平。继续抓好住房公积金归集扩面工作。深入推进社会养老服务体系建设，为困难家庭的失能老人和高龄老人提供居家养老服务支持。整合各项社会救助制度，建立“一门受理、协同办理”机制。大力发展残疾人事业，做好全国第九届残运会暨第六届特奥会眉山分会场工作。

26.实现充分就业创业。坚持创业带动就业，落实好促进创业、就业的各项优惠政策，鼓励高校毕业生、返乡人员、失地农民、退伍军人自主创业和灵活就业。完善就业公共服务体系，实施提升劳动者就业创业能力专项行动，加大就业困难人员帮扶力度，全年城镇新增就业2.4万人。

27.构建文化服务体系。深入推进“文化立市”战略，广泛开展群众性文化创建活动，加快构建现代公共文化服务体系。深化“眉山东坡大舞台”品牌文化活动，推进数字电视、广播村村响、农村公益电影放映工程。深入实施全民健身计划，加快“五馆一中心”、中国竹编艺术城建设。积极发展文化产业，大力促进文化体育消费。推进传统媒体与新媒体融合发展，繁荣艺术创作，加强文化遗产保护和利用。

28.全面发展社会事业。大力推进学前教育规范发展、义务教育均衡发展、普通高中优质发展。大力发展现代职业教育，培养高素质劳动者和技能型人才。改善办学条件，新建、改扩建校舍5万平方米，加快青衣小学、青衣中学建设。加快市医院二期工程、市妇保院二期工程建设和市中医院整体迁建。加强疾病预防控制、妇幼卫生保健、卫生监督执法三大公共卫生体系建设。提高计划生育服务管理水平。积极发展中医药事业。扎实抓好妇女儿童、关心下一代、民族宗教、外侨台、地方志、档案等工作。做好“十三五”规划编制工作。

八、突出社会和谐，提升治理效力

稳定才能发展，平安才能和谐。我们将坚持以法治为引领，不断创新社会治理方法，提高社会治理水平，确保社会和谐稳定。

29.改进社会治理方式。深入实施依法治市纲要，扎实开展法治示范创建活动，加快建设覆盖城乡的公共法律服务体系。继续推进社会治理创新，巩固深化网格化服务管理工作，抓好特殊人群服务管理改革综合试点，完善提升社会稳定风险评估、“大调解”和“诉非衔接”机制。加强城乡社区建设，健全基层综合服务管理平台，完善村务公开、居务公开和民主管理制度。支持工会、共青团、妇联、工商联等人民团体参与社会治理。

30.健全公共安全体系。树立新型安全观，抓好国家安全、保密和人民防空工作，强化国防动员和后备力量建设，争创双拥模范城。扎实推进“平安眉山”建设，建立健全社会治安立体化防控机制，挂牌整治治安混乱地区，依法严厉打击各类违法犯罪活动。建立健全安全生产责任体系，夯实基层基础，坚决遏制重特大事故发生；加强食品药品安全监管执法，确保“舌尖上的安全”；健全地质灾害防治体系，做好应急、防震减灾、气象、测绘等工作，提升公共安全和防灾救灾减灾能力。

努力建设法治政府

深入贯彻依法治国基本方略，坚持崇尚法治、厉行法治，把政府工作全面纳入法治轨道，加快建设职能科学、权责法定、执法严明、公开公正、廉洁高效、守法诚信的法治政府。

坚持依法履职。忠实履行宪法和法律赋予的职责，更加自觉地运用法治思维和法治方式履行职责、服务群众。抓紧建立法无禁止皆可为的“负面清单”、法无授权不可为的“权力清单”、法定职责必须为的“责任清单”，推进政府机构、职能、权限、程序、责任法定化。全面正确履行政府职能，更加重视公共服务和社会管理。进一步健全四级政务服务体系，加快政务服务平台和公共资源交易平台规范化建设。

坚持依法决策。严格执行公众参与、专家论证、风险评估、合法性审查、集体讨论决定的重大行政决策法定程序，提高政府依法决策水平。建立行政机关内部重大决策合法性审查机制。健全政府常务会议和部门办公会议学法制度，完善政府法律顾问工作制度。建立决策跟踪评估制度，落实行政决策终身责任追究制度及责任倒查机制。

坚持依法行政。认真执行人大决议、决定，自觉接受人大、政协和社会监督。深化行政执法体制改革，大力推进综合执法，减少行政执法队伍种类。建立健全行政裁量权基准制度，规范行政执法行为。坚持以公开为常态、不公开为例外，全面推进政务公开、政府信息公开和政府网站建设。狠抓政府廉政建设和作风建设，加强行政权力行使监督，持之以恒反四风、转作风、树新风，坚决治理“庸懒散浮拖”等不良现象，努力让人民群众从地方行政和公务人员身上感受到新风正气。

各位代表！信心在勇气就在，努力在成功就在。只要顶住经济下行的压力，只要闯过转型升级的关口，只要守住风险防控的底线，眉山的发展前景将更加光明。让我们在省委、省政府和市委的坚强领导下，凝聚起全市人民的智慧和力量，奋发有为、苦干实干，为建设富裕美好眉山而努力奋斗！
